# Psychophysiological and Metabolomics Responses of Adults during Horticultural Activities Using Soil Inoculated with *Streptomyces rimosus*: A Pilot Study

**DOI:** 10.3390/ijerph191912901

**Published:** 2022-10-08

**Authors:** Seon-Ok Kim, Min Ji Kim, Na-Yoon Choi, Jin Hee Kim, Myung Sook Oh, Choong Hwan Lee, Sin-Ae Park

**Affiliations:** 1 Department of Bio and Healing Convergence, Graduate School, Konkuk University, Seoul 05029, Korea; 2 Department of Bioscience and Biotechnology, Konkuk University, Seoul 05029, Korea; 3 Department of Biomedical and Pharmaceutical Sciences, Graduate School, Kyung Hee University, Seoul 02447, Korea; 4 MetaMass Corp., Seoul 05029, Korea; 5 Department of Systems Biotechnology, Konkuk University, Seoul 05029, Korea

**Keywords:** 2-methylisoborneol, brain-derived neurotrophic factor, C-reactive protein, electroencephalogram, gardening, geosmin, horticultural therapy, metabolite profiling, soil microorganism, volatile compounds

## Abstract

This study compared the physiological effects at a metabolomics level with autonomic nervous system responses in adults during soil mixing activities, based on the presence or absence of *Streptomyces rimosus* in the soil. Thirty adult participants performed soil mixing activities for 5 min using sterilized soil with culture media and *Streptomyces rimosus*, respectively. Blood samples were drawn twice from each participant after each activity. Electroencephalograms were measured during the activity. Serum metabolites underwent metabolite profiling by gas chromatography, followed by multivariate analyses. Serum brain-derived neurotrophic factor and C-reactive protein levels were measured by Enzyme-Linked Immunosorbent Assay. Soil-emitted volatile organic compounds were identified via solid-phase microextraction and gas chromatography–mass spectroscopy, followed by multivariate analyses. The volatile compound analysis revealed that the terpenoid and benzoid compounds, geosmin, and 2-methylisoborneol were greater in soil with *Streptomyces rimosus*. Serum metabolomics revealed that the treatment group (soil inoculated with *Streptomyces rimosus*) possessed relatively higher levels of serotonin compared to the control group (soil mixed with culture media), and serum C-reactive protein levels were significantly lower in the treatment group. In the treatment group, the electroencephalogram revealed that alpha band activity of the occipital lobe increased. This study concludes that *Streptomyces rimosus* soil contact can positively affect human metabolic and autonomic reactions. Therefore, this pilot study confirmed the possible role of soil microorganisms in horticultural activities for psychophysiological effects in humans.

## 1. Introduction

Soil is the most extensive reservoir of natural microorganisms on the planet, and contact with beneficial soil microorganisms has been reported to have a positive effect on human health. *Streptomyces* is a soil microorganism, a type of actinomycete, and some species are used as raw materials for antibiotics to develop various drugs [1]. In particular, *Streptomyces rimosus* (*S. rimosus*) is best known as the primary source of the tetracycline class of antibiotics—most notably, oxytetracycline—which have been widely used against many Gram-positive and Gram-negative pathogens and protozoan parasites [2]. 

Geosmin and 2-methylisoborneol (2-MIB) are secondary metabolites produced by *S. rimosus*, and they are known as sources of the earthy–musty smell [3]. Geosmin and 2-MIB belong to the group of terpenoid compounds and are synthesized by terpene synthetase, but they have different molecular formulas and structural arrangements [4]. In a previous study, as a result of inhalation of geosmin and 2-MIB in adults, cortical beta activity decreased and alpha activity increased, resulting in stabilization of brain waves [4]. 

Such positive olfactory stimulation has advantages such as reducing mental stress and improving relaxation and cognitive function [5,6]. When a fragrance molecule is inhaled through the human nose, the inhaled fragrance molecule binds to the olfactory receptor, activates the olfactory receptor cell, transmits an electrical signal to the olfactory bulb through the neural network, and stimulates the hypothalamus and cerebral cortex through the process through the limbic system of the brain [7,8,9]. Odor molecules play an important role in human behavior such as emotions, thoughts, and memory by influencing spontaneous brain function through the olfactory system [10]. Aromatic compounds of plants have been reported to have various physiological effects on psychological aspects, emotional stability, and the nervous system [11,12,13]. However, there are still few studies on the effect of olfactory stimulation through soil and soil microorganisms on humans.

One of these studies was conducted to identify the healing effect of contact with specific soil microorganisms. In that study, EEG analysis and serum metabolite profiling were performed to determine the psychophysiological effects of horticultural activities using soil inoculated with *Mycobacterium vaccae* (*M. vaccae*), a soil microorganism belonging to Actinomycetales [14]. The study demonstrated that contact with the soil microorganism significantly affected the brain, metabolomics response, and autonomic nervous activities, with these changes inducing a state of relaxation in humans.

Although it is generally known that the earthy–musty smell has an emotional stabilization effect, there have been few studies on the interaction between soil microorganisms and humans, and the healing mechanism has not been clearly revealed. It is necessary to study the effects of interactions among soil, soil microorganisms, and humans to clearly understand the therapeutic mechanisms of horticultural activity intervention and nature-based therapy. Therefore, this study measured the effects of a horticultural activity in direct contact with soil microorganisms on the psychological and physiological responses of humans, according to the presence or absence of *S. rimosus* in the soil. To assess the feasibility of the role of *S. rimosus* in the horticultural activity intervention, we designed this pilot study to identify the effects of olfactory stimuli on humans, focusing on the volatile organic compounds (VOCs) that *S. rimosus* develops. 

## 2. Materials and Methods

### 2.1. Participants 

This study was conducted with 30 adults in their 20s and 50s (11 males, 19 females, average age 28.6 ± 8.4 years old), who were recruited using the convenience sampling method. Flyers with research information were distributed to apartments and libraries in Gwangjin-gu, Seoul, Korea. Based on a previous study on the psychophysiology of horticultural activity [15,16], the number of participants was set to 30 in the crossover study design. 

Based on a previous study that showed that brain activity differs according to the dominant hand, only right-handed participants participated in this study [17]. Healthy adults without any current disease [18] and with no olfactory dysfunction or respiratory disease were considered. In addition, the naturally occurring physiological effects were eliminated by fasting and smoking cessation for 2 h before the experiment. Before proceeding with the experiment, the contents of the study and precautions were explained, and written consent was obtained before participation. Age, gender, height, weight, and body mass index (ioi 353; Jawon Medical Co., Ltd., Gyeongsan, Korea) were recorded to collect demographic information on the participants. Participants received an amount equivalent to USD 12 as an incentive for completing the experiment. The present study was approved by the Bioethics Committee of Konkuk University (7001355-202106-HR-442). 

### 2.2. Selection of the Streptomyces Strain

**Figure 1:**
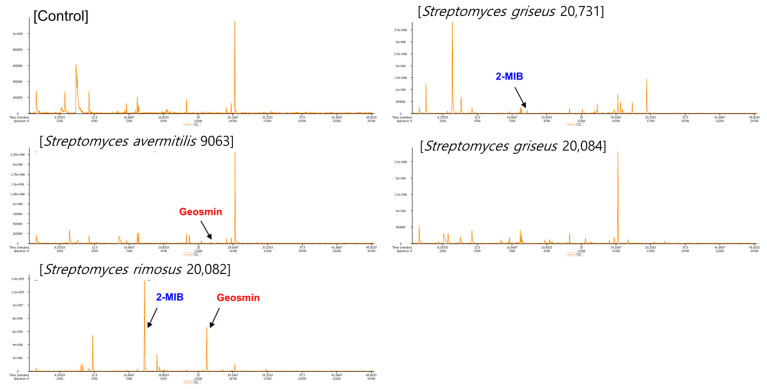
Results of headspace solid-phase microextraction gas chromatography–time-of-flight–mass spectrometry datasets for various *Streptomyces* strain samples.

The strains *Streptomyces griseus* (*S. griseus*) KACC 20084, *Streptomyces griseus* (*S. griseus*) KACC 20731, and *S. rimosus* KACC 20082 were procured from the Korean Agricultural Culture Collection (KACC), Republic of Korea. The strain *S. avermitilis* KCTC 9063 was supplied by the Korean Collection for Type Cultures (KCTC), Republic of Korea. Each microorganism was cultivated on tryptic soy broth for four days at 27 °C with shaking (250 rpm). All *Streptomyces* strains were analyzed for the production of geosmin and 2-MIB using SPME GC–MS (Table S1). For preparation of the VOCs, an aliquot of 80 μL of bacterial suspension was inoculated into a 20 mL clear vial with a PTFE septum and a screw cap containing 8 mL of tryptic soy broth. Inoculated and uninoculated broths were incubated for four days at 27 °C and then subjected to volatile profiling via HS-SPME GC–TOF–MS. Among these isolates, *S. rimosus* KACC 20082 was selected for further study because it produced both geosmin and 2-MIB (Figure 1). The *m*/*z* values of the most abundant ion fragments were 95 for 2-MIB and 112 for geosmin.

### 2.3. Preparation of the Soil Sample

*S. rimosus* KACC 20082 was cultivated on tryptic soy broth for four days at 27 °C with shaking (250 rpm). For soil sample preparation, soil samples were autoclaved at 121 °C for 15 min. The sterile soil (1.5 g) was mixed with 2.5 mL of sterile water, a tryptic soy broth, and four-day cultured *S. rimosus* strain for three days at 27 °C to obtain various types of VOCs. After incubation, the samples were transferred to a gas chromatography–time-of-flight–mass spectrometry (GC–TOF–MS) instrument to analyze the VOCs.

### 2.4. Experimental Environment

**Figure 2:**
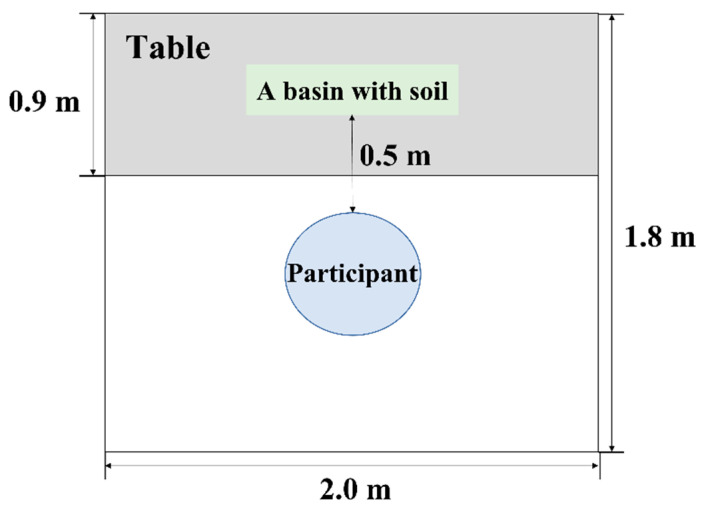
Room arrangement of the experiment.

This study was conducted in the experimental space (180 cm × 200 cm) of Konkuk University, Seoul, Korea (Figure 2). The environmental conditions of the space during the experiment were as follows: temperature 27.3 ± 3.0 °C (O-257; DRETEC Co., Ltd., Saitama, Japan), humidity 43.3 ± 15.8% (O-257; DRETEC Co., Ltd.), and illuminance 2097.9 ± 1076.8 Lux Meter (ST-126; SINCON, Bucheon, Korea). To minimize visual stimulation during the experiment, white cardboard was laid out in front of the desk and ivory-colored curtains were placed on both sides. Participants were placed facing the center of the desk.

### 2.5. Experimental Protocol

**Figure 3:**
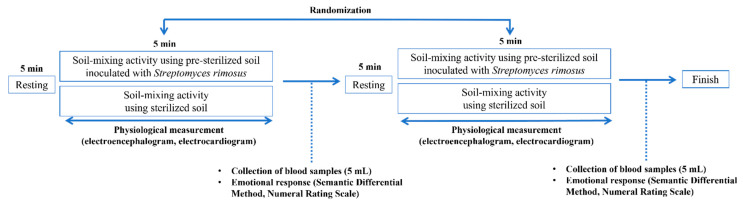
Experiment protocol. EEG: electroencephalogram; SDM: comparisons via the Semantic Differential Method; Stress: subjective stress evaluation (NRS: numeral rating scale).

This study was a single-blinded randomized crossover trial. Participants performed the experimental protocol presented in Figure 3 to investigate the effect of a soil mixing activity using soil containing *S. rimosus* on the physiological response of adults. Before performing the soil mixing activity, participants were asked to face forward and rest for 5 min. Then, they mixed the soil in the basin for 5 min. Thereafter, 5 mL of blood was collected from the participants for metabolite analysis after each soil mixing activity. In addition, the Semantic Differential Method (SDM) and subjective stress evaluation using a numeral rating scale (NRS) were performed to evaluate subjective emotional states. After the first trial, the participants performed the other trial after a 5 min rest period, and the trial order was randomly assigned. The total duration of the experiment was up to 40 min per participant.

Sterilized peat moss (2000 mL), perlite (800 mL), and water (200 mL) were mixed in the experimental group soil and cultured for 3 days using 50 mL of *S. rimosus* medium. For the control soil, 50 mL of a culture solution without microorganisms was mixed with the same material.

### 2.6. Measurement

#### 2.6.1. Psycho-Physiological Measurement 

To compare the psychophysiological responses of participants when performing soil mixing activities according to the presence or absence of *S. rimosus*, the responses were divided into physiological data and psychological data. In this study, EEG was measured using a wireless dry EEG device (Quick-8; Cognionics, San Diego, CA, USA). The device uses dry electrodes to minimize the risk of electrical stimulation, and it has been certified for safety by the European Commission and the Federal Communications Commission [19]. 

**Figure 4:**
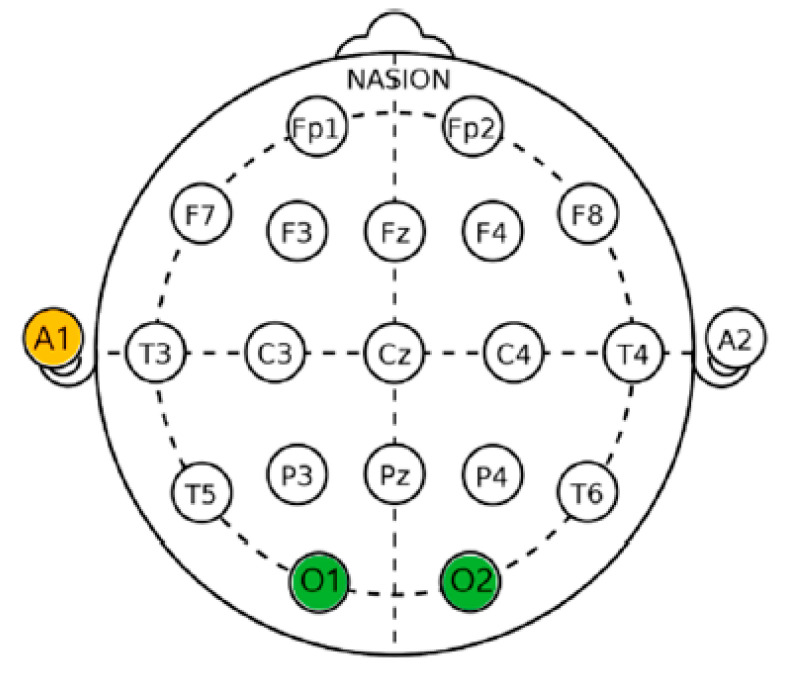
International electrode arrangement [20].

Data were collected by amplifying the electrical signal measured by attaching a dry electrode to the scalp. Electrode application complied with the international 10–20 electrode arrangement system [20]. A reference electrode was attached to the left earlobe (A1). In this study, EEG monitoring was performed at O1 (left occipital cortex) and O2 (right occipital cortex) (Figure 4). It has been reported that EEG can improve our understanding of brain activity and human central nervous system activity through olfactory stimulation. 

In addition, previous studies showed that soil microorganism *M. vaccae* was effective in reducing depression by increasing serotonin [21]; serotonin acts as a major neurotransmitter in the occipital lobe, which also regulates human emotions and mood. Therefore, in this study, the occipital cortex was selected and analyzed to investigate the effects of soil mixing activity on human emotional mood and physiological changes according to the presence or absence of *S. rimosus*.

The Semantic Differential Method (SDM) was developed as a questionnaire to measure subjective emotional state with adjectives [22]. The items consist of a total of three items: ‘comfort–uncomfortable’, ‘natural–artificial’, and ‘comfort–awakening’. A higher score reflects a positive mood state. To evaluate the stress of participants, we used a 0 to 10 NRS. A higher score reflects a positive mood state. The higher the score, the higher the stress state. 

#### 2.6.2. Soil Sample Extractions and Analysis of VOCs by GC–MS

The extraction and analysis of VOCs followed that by Lyu et al. [23] and Lee et al. [24] with few modifications. Headspace solid-phase microextraction (HS-SPME) was performed for three biological replicates of soil treated with distilled water (*S*), culture media (*SM*), or *S. rimosus* KACC 20082 (*SS*) to obtain VOCs. Each soil sample (1.5 g) was mixed with 2.5 mL of distilled water, culture media, or the *Streptomyces* strain. The sample mixtures were transferred into 20 mL SPME vials and incubated for 3 days. For profiling of the VOCs, each vial was maintained at 50 °C for 30 min. After this, VOCs from the headspace of each vial were collected using carboxen/polydimethylsiloxane/divinylbenzene (CAR/PDMS/DVB)-coated SPME fibers (75 μm; Supelco Inc., Sigma-Aldrich, St. Louis, MO, USA). The fiber was exposed to soil samples at 50 °C for 30 min with 250 rpm for emission of the VOCs. The fiber containing the volatiles was then automatically injected into an Agilent 7890A GC system (Agilent Technologies, Palo Alto, CA, USA) for desorption for 5 min. After desorption, the oven temperature was initially held at 40 °C for 3 min, further increased to 150 °C at a rate of 4 °C/min, held at 150 °C for 1 min, and further increased to 250 °C for 2 min. The carrier gas used was helium with a flow velocity of 1.0 mL/min, and the transfer line temperature was 260 °C. Mass spectra were scanned from 50 to 500 *m*/*z* at a rate of 10 scans.

#### 2.6.3. Measurement of Blood Metabolites

Blood samples were collected to measure changes in brain-derived neurotrophic factors. A trained professional nurse collected a blood sample (5 mL). The collected sample was stored in an ice pack, then maintained at room temperature for 20 min, then centrifuged at 3000 rpm for 10 min to separate serum samples. After that, aliquots were stored in a deep freezer at −70 °C. The serum extraction procedure and GC–TOF–MS analysis followed that in our previous research [25]. Each human serum (200 μL) was extracted with cold methanol (1 mL) and 10 μL of an internal standard (2-chlorophenylalanine, 1 mg·mL^−1^) using a mixer mill (MM400; Retsch, Haan, Germany) at a frequency of 30 Hz for 10 min, with sonication for another 10 min. After homogenization, the suspension was stored at 20 °C for 60 min. It was then centrifuged at 13,250× *g* for 10 min at 4 °C (Zentrifugen Universal 320; Hettich, Tuttlingen, Germany). The supernatant was filtered through a 0.2 μm polytetrafluorethylene (PTFE) filter (Chromdisc, Daegu, Korea). The filtered samples were dried completely using a speed vacuum concentrator (Biotron, Seoul, Korea). The final concentration of each sample was adjusted to 10 mg·mL^−1^ for the mass spectrometry (MS) analysis. The derivatized samples (1 μL) were injected into the GC–TOF–MS instrument in the splitless mode. The *m*/*z* value of the most abundant ion fragments was 202 for serotonin. The analytical samples were randomized in each block to reduce the effects of systematic errors.

#### 2.6.4. Measurement of Brain-Derived Neurotrophic Factor (BDNF) and C-Reactive Protein (CRP) Levels in Serum

The serum was stored at −80 °C until assay, and only hemolyzed samples of <50 mg/dL were used according to the hemolysis reference palette. Serum BDNF and CRP levels were measured by Enzyme-Linked Immunosorbent Assay (ELISA) according to the manufacturer’s instructions (AbCAM, Cambridge, UK).

### 2.7. Data Analysis

The measured EEG data were analyzed using the Bio-scan (Bio-Tech, Daejeon, Korea) program. The collected EEG raw data were analyzed using power spectrum analysis to identify the spectral edge frequency as 50% of alpha (ASEF50) [26]. ASEF50 is an index indicating brain comfort and is calculated as a band corresponding to 50% of the alpha (8 to 13 Hz) power. Data were collected by measuring the average of the EEG during the experiment, and a brain map program (Biotec Analysis Software, Daejeon, Korea) was used.

MS data processing and multivariate statistical analysis were performed as previously described [25]. For MS data processing, raw data obtained from GC–TOF–MS were converted to a netCDF (*.cdf) format using LECO ChromaTOF software (version 4.44, LECO Corp., St. Joseph, MI, USA). Subsequently, the peak selection, retention time, and peak alignment were determined using MetAlign software (RIKILT-Institute of Food safety, Wageningen, The Netherlands). The results of alignment data were exported to a Microsoft Excel file. Multivariate statistical analysis was performed using SIMCA-P+ ver. 12.0 software (Umetrics, Urea, Sweden). Principal component analysis (PCA), partial least squares discriminant analysis (PLS-DA), and orthogonal partial least squares discriminant analysis (OPLS-DA) were performed to compare different VOCs and serum metabolites. The significance levels of the PLS-DA and OPLS-DA models were defined by analysis of variance of cross-validated predictive residuals (CV-ANOVA) using the SIMCA-P+ program. The significantly discriminant metabolites were selected on the variable importance in the projection (VIP) values of the PLS-DA and OPLS-DA models. The selected metabolites were identified through comparing mass spectra (MS) and their retention time to the available databases, such as the National Institute of Standards and Technology (NIST) database (version 2.0, 2011, FairCom, Gaithersburg, MD, USA), the Human Metabolome Database (HMDB; http://www.hmdb.ca/ (accessed on 27 February 2022)), and our in-house library of standard compounds.

The processed EEG data, SDM, and NRS for each stimulus were analyzed via paired *t*-test, which was performed using the SPSS (Version 25 for Windows; IBM, Armonk, NY, USA) program. For the demographic information collected, descriptive statistics were obtained for the mean, standard deviation, and percentage of each item using Microsoft Excel (Office 2007; Microsoft Corp., Redmond, WA, USA).

The serum BDNF and CRP levels were expressed as the mean ± SD using Graph Pad Prism 8.0.1 software (Graph Pad software Inc., San Diego, CA, USA). The results were analyzed statistically via paired *t*-test. Differences with a p-value less than 0.05 were considered statistically significant. 

## 3. Results

### 3.1. Demographic Information 

The participants in this study were 30 adults in their 20s to 50s (11 males and 19 females; average age: 28.6 ± 8.4 years), with characteristics as shown in Table 1.

### 3.2. Psycho-Physiological Responses

In the EEG comparison during the soil mixing activity (based on the presence or absence of *S. rimosus* in the soil), the ASEF50 of the right occipital lobe was significantly higher during the soil mixing activity including *S. rimosus* (*p* < 0.01; Table 2). The results analyzing the difference in blood pressure and pulse after mixing the soil in the presence and absence of *S. rimosus* were not significant.

**Figure 5:**
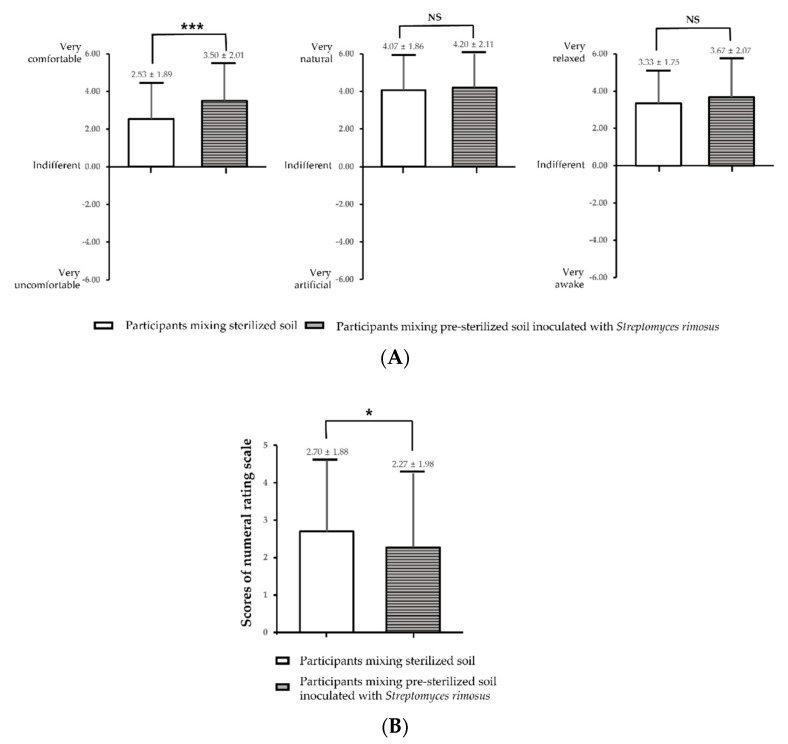
(**A**) Comparisons of the Semantic Differential Method (SDM) for each soil mixing activity. (**B**) Subjective stress evaluation. NRS, numeral rating scale. * *p* < 0.05, *** *p* < 0.001, ^NS^ *p* > 0.05 by the paired *t*-test, respectively. Values are the mean ± SD.

As a result of the SDM evaluation based on the soil mixing activity according to the presence of *S. rimosus* in the soil, a relatively high “comfortable” (*p* < 0.001) was shown when the soil mixing activity was performed in the soil with *S. rimosus* (Figure 5). In addition, as a result of evaluating subjectively felt stress using an NRS on a scale of 0 to 10, the stress level was significantly reduced after the soil mixing activity in soil containing *S. rimosus* compared to that in control soil without *S. rimosus* (*p* < 0.05).

### 3.3. Volatolome Profiling of Soil Samples

**Figure 6:**
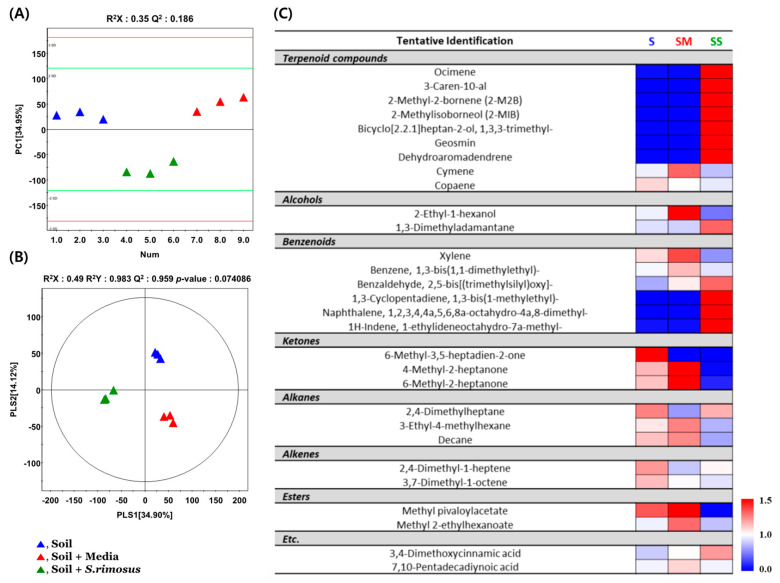
(**A**) Principal component analysis (PCA) and (**B**) partial least square discriminant analysis (PLS-DA) score plot derived from SPME–GC–TOF–MS datasets for various soil samples. Symbols: soil treated with distilled water (S, ▲); soil treated with culture media (SM, ▲); soil inoculated with *S. rimosus* (*SS*, ▲). (**C**) Heat map analysis for the relative abundance of different volatile organic compounds (VOCs) (VIP > 0.7, *p* < 0.05) derived from the GC–TOF–MS analysis. The colored squares (blue to red) indicate fold changes that are normalized by the average of each metabolite.

Discrepancies in VOCs were identified in various soil samples, including those from S, SM, and SS. These were evaluated via multivariate analysis of the SPME–GC–TOF–MS data set. As can be seen in Figure 6, the PCA score plots based on the SPME–GC–TOF–MS data showed distinct differences from the different soil samples according to PC1 (34.95%) and PC2 (14.12%, Figure 6A). The statistical parameters of PLS-DA models were verified with R^2^X (0.49), R^2^Y (0.983), and Q^2^ (0.959), indicating the fitness and prediction accuracy of the model at *p*-values (*p* < 0.05) obtained through cross-validation (Figure 6B). PLS-DA model analysis confirmed that the SS sample was separated from the remaining treatment group by PLS1 [34.90%], while the S sample was separated from the rest of the treatment group by PLS2 [14.12%]. Therefore, although the PLS-DA (group information included, biased) model was not significant, different metabolites were selected based on the PLS-DA model (VIP > 0.7). As a result of analyzing significantly different VOCs between soil samples, a total of 29 VOCs were identified (9 terpenoid compounds, 2 alcohols, 6 benzenoids, 2 alcohols, 3 ketones, 3 alkane, 2 alkenes, 2 esters, and 6 others). The relative contents of the discriminant metabolites are displayed in a heat map (Figure 6C). According to the heat map analysis, most terpenoids and benzenoids showed relatively high content in the *SS* samples. Also, geosmin and 2-MIB, selected as target VOCs, were uniquely detected in the *SS* sample and showed relatively high contents (*p* < 0.05).

### 3.4. Metabolite Analysis and Correlation Analysis of Serum Metabolites after the Effect of the Soil Mixing Activity

**Figure 7:**
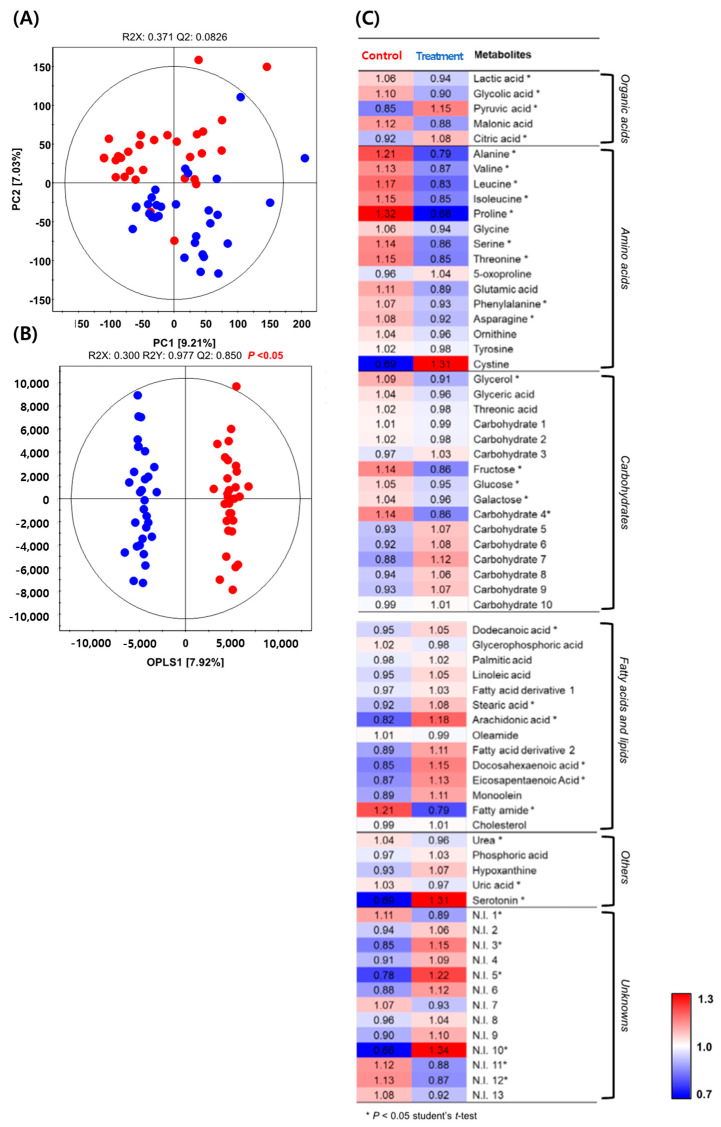
(**A**) Principal component analysis (PCA) and (**B**) partial least square discriminant analysis (PLS-DA) score plot derived from GC–TOF–MS datasets for serum samples. Symbols: control (soil treated with SM, ●); treatment (*SS*, ●). (**C**) Heat map analysis for the relative abundance of different serum metabolites (VIP > 1.0) derived from GC–TOF–MS analysis. The colored squares (blue to red) indicate fold changes that were normalized by the average of each metabolite. * Significantly different metabolite between the control and treatment groups (*p* < 0.05, Student’s *t*-test).

Based on the results of soil variation according to VOCs, metabolite profiling of serum samples was performed to confirm the metabolite levels after the soil mixing activity for both SM and *SS* and to clearly understand how they were affected. As a result, the PCA score plot for the serum data set showed an unclear cluster between the OPLS-DA control group (SM) and the experimental group (*SS*) (Figure 7A), whereas the OPLS-DA score plot showed a clear difference between SM and *SS* (Figure 7B). According to the OPLS-DA model, discriminant metabolites were selected between the control and treatment groups with VIP values of >1.0. A total of 68 metabolites were identified (5 organic acids, 15 amino acids, 14 fatty acids and lipids, 5 other, and 13 unknowns) (Table S2). For visualization of the various metabolites, all were plotted on a heat map (Figure 7C). 

According to the heat map analysis, most of the fatty acids, lipids, and others, except for the glycerophosphoric acid, oleamide, fatty amide, cholesterol, urea, and uric acid, were relatively higher in *SS* samples than in the control group. In particular, serotonin, considered as the major neurotransmitter involved in human emotions and mood, showed significantly higher contents in the *SS* sample (*p* < 0.05). 

### 3.5. Effect of BDNF and CRP Level in Serum after Each Soil Mixing Activity 

According to the previous results that the soil mixing activity with *S. rimosus* changed the EEG and increased the serotonin level, we additionally analyzed mood-related serum factors, BDNF and CRP. BDNF is known to regulate neuronal survival and is associated with psychological dysregulation such as anxiety and depression [27,28]. Also, serum CRP is used as an inflammatory biomarker, and a high CRP level is related to depression and euthymia [29,30,31]. 

**Figure 8:**
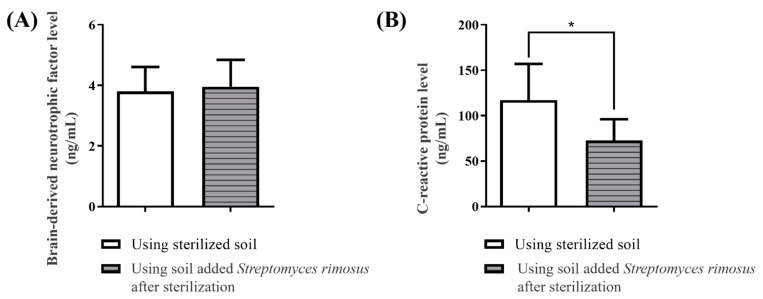
Results of (**A**) BDNF and (**B**) CRP levels in serum after the soil mixing activity with or without *S. rimosus* (n = 16 per group). Data were analyzed via paired *t*-test. * *p* < 0.05 compared to the sterilized soil group. Values are the mean ± SD.

In the analysis, the BDNF level in the serum was unchanged after the soil mixing activity with or without *S. rimosus* (Figure 8A). However, the CRP level in the serum significantly decreased after the soil mixing activity including *S. rimosus* (*p* < 0.05; Figure 8B).

## 4. Discussion

Various effects in psychological and physiological aspects obtained through interaction with nature have been reported [14], but there have been few studies on the role of soil and soil microorganisms. In this study, we investigated the effects of contact with *S. rimosus* using metabolic approaches and various psychophysiological parameters, focusing on VOCs derived by *Streptomyces* soil microorganisms. The result of this study showed that horticultural activity using soil containing *S. rimosus* causes psychophysiological changes in human brain activity, metabolomics response, and serum BDNF and CRP.

First, in order to select a strain to be used for the experiment, candidate strains based on previous studies were selected (Control; SA: *S.aversmitilis* KCTC 9063; SG1: *S.griseus* KACC 20084; SG2: *S.griseus* KACC 20731; SR: *S. rimosus* KACC 20082) and analyzed via SPME. Among them, geosmin and 2-MIB, the target VOCs, were detected in the *S. rimosus* strain and showed relatively high contents (*p* < 0.05). The selected strain, *S. rimosus* 20082, was inoculated into the soil (*SS*), and the effects of VOC profiling and a soil mixing activity with various soil sample samples (S, SM) were investigated. 

As a result of VOC analysis of various soil samples, most of the VOCs were found to be higher in *S.* *rimosus* samples than in other soil samples. Some VOCs of terpenoid and benzoid series and the target VOCs geosmin and 2-MIB showed relatively high contents in the *SS* soil group. It has been reported that these VOCs from soil play an important role in relieving inflammation and stress, improving sleep disorders, and regulating the immune system when humans are exposed to them [32,33]. In addition, a study of soil microorganism *M. vaccae*, which was found to be effective in improving respiratory diseases and cognitive function, found that the immune system tends to activate and increase serotonin, which can affect emotional stability and behavioral responses [33].

In addition, to understand the effect of the soil mixing activity, EEG, serum metabolite analysis, and subjective emotional evaluation were performed between the experimental group (soil with *S. rimosus*) and the control group (soil without *S. rimosus*). As a result, the effect of the soil mixing activity on human metabolic and autonomic responses was found to differ according to the presence or absence of *S. rimosus* microorganisms in the soil.

As a result of the EEG, ASEF50 of the right occipital lobe was significantly higher in the treatment group (soil with *S. rimosus*) than in the control group (soil without *S. rimosus*) (*p* < 0.01). ASEF50 is the frequency of the point occupying 50% of the 8–13 Hz range, which is the alpha wave band, in the power spectrum graph. Increased cortical alpha activity indicates brain comfort and is associated with a clear wakefulness state in the brain just prior to concentration. Also, inhalation of 2-MIB (a major odor molecule in soil), which was placed as a target VOC, significantly increases the rapid alpha band among human brain waves.

In the olfactory process, fragrant molecules attach to the olfactory receptor cilia in the olfactory epithelium located in the nasal cavity, activate guanine nucleotide binding protein coupled receptors, and generate electrical signals [34]. Thereafter, electrical signals are transmitted to the brain by olfactory sensory neurons through the olfactory bulb and higher olfactory cortex to modulate brain functions, including memory, thoughts, and emotions [35]; furthermore, they act on the neuroendocrine system, neurotransmitters, and neuromodulators, influencing psychological behavior and body function [6]. Many studies have explained that inhalation of fragrances has a significant effect on brain function and various psychophysiological parameters such as blood pressure, muscle tension, pupil dilation, skin temperature, pulse rate, and brain activity, because fragrance compounds can cross the blood–brain barrier and interact with receptors in the central nervous system [10,36,37,38]. Therefore, these results suggest that the olfactory stimulation caused by soil-derived VOCs also affected the central nervous system and cerebral cortex during the soil mixing activity with the soil containing *S. rimosus*, and it is thought that it would induce a pleasant and clear arousal state of the brain. It is also thought that such a positive psychophysiological response had a positive effect on the subjective emotional states of the participants. 

A previous study reported that serum metabolites can be affected by physical activity [14]. Serum metabolomics showed that the *S. rimosus* treatment group (*SS*) displayed relatively higher levels of fatty acids, lipids, and other molecules compared to the control group (S) (Figure 7C). However, most of the organic acids and amino acids were relatively higher in the control group. In particular, cystine and serotonin levels in the *SS* group were significantly higher than in the control group (Figure 7C). Generally, serotonin is known as an important neurotransmitter involved in the regulation of numerous biological and behavioral functions in the body, playing a crucial role in psychological processes in the central nervous system, including cognition and pain sensitivity [25,39]. Silber et al. [40] reported that serotonin has been correlated with emotional and motivational aspects of human behavior, such as anxiety disorders and depression. A previous study reported that psychological disorders such as depression have been correlated with serotonin contents, which showed decreasing patterns [25]. Cystine, which is the major form of cysteine under physiological conditions, is more stable in blood plasma than the free amino acid cysteine. In the cells, cystine is promptly converted to cysteine, which is an essential substrate for the synthesis of biomolecules such as lipids, proteins, glutathione (SGH), and Coenzyme A [41,42]. Moreover, cysteine supports various processes, including detoxification and steroid metabolism [43].

In particular, several 5-HT (serotonin) receptors are expressed highly by most excitatory neurons in the occipital cortex [44,45]. Therefore, this positive correlation may have appeared as an association between the activity of the occipital cortex and the activity of the serotonin system.

In a previous study, it was reported that BDNF regulates aging and brain activity in the hippocampus and surrounding areas [46,47]. Also, BDNF has been found to be associated with brain and psychological disorders such as memory dysfunction, depression, and anxiety. As a result of our serum analysis, the soil mixing activity in the presence or absence of *S. rimosus* did not change the amount of BDNF. On the other hand, the CRP level decreased after the soil mixing activity containing *S. rimosus*. CRP is a pentagonal protein produced in the liver, and CRP levels in the serum increase due to acute and chronic inflammatory reactions [29]. For this reason, CRP is a major inflammatory factor used as an inflammatory biomarker [48]. It has also been reported that high CRP levels are associated with psychological disorders such as depression and bipolar mood disorders [30,31]. Accordingly, the current results imply that soil mixing activities including *S. rimosus* can reduce inflammation and alleviate elevated serotonin levels in a short period of time; they may consequently cause psychophysiological changes, including being comfortable or relieved from stress. 

With similar results, contact with soil microorganism *M. vaccae* has been shown to have positive effects on human metabolic and autonomic reactions. A soil mixing activity with *M. vaccae* increased serum organic acids, decreased fatty acids, and stabilized brain activity and the autonomic nervous system [14]. Therefore, our results are in line with such findings of the effects of soil microorganisms on psychophysiological aspects. The difference from the previous study is that soil microorganisms were selected by selecting target VOCs investigated through previous studies. Thus, it was possible to take one step closer to identifying the mechanism of the healing effect of contact with soil microorganisms. 

## 5. Conclusions

In conclusion, the soil mixing activity with *S. rimosus* showed an increased level of serum serotonin and decreased levels of CRP. In addition, the alpha activity in high-frequency bands of the occipital cortex increased, and the positive subjective emotional state score also increased. These results suggest that contact with *S. rimosus* and exposure to VOCs derived from *S. rimosus* had a positive effect on human mental health. The effects and mechanisms of VOCs generated by soil microorganisms on human mental health need to be continuously studied; furthermore, the benefits of nature such as those from plants, soil, and soil microorganisms must be continuously utilized.

The limitations of this pilot study were that the sample size was small and that it was conducted within a controlled study design. Therefore, it is difficult to generalize the results of this study, and there is a limitation in not being able to fully explain the effect of an actual long-term horticultural activity intervention. Therefore, it would be interesting to investigate the effects of contact with soil microorganisms on psychophysiological aspects through long-term horticultural activity interventions in a larger sample size in a follow-up study.

## Figures and Tables

**Table 1:** Descriptive characteristics of the participants (N = 30).

Variable	
Gender	% (N)
Male	36.7 (11)
Female	63.3 (19)
	Mean (SD)
Age (years)	28.6 (8.4)
Height (cm)	168.0 (8.4)
Body weight (kg)	62.9 (12.6)
Body mass index ^1^ (kg·m^−2^)	22.1 (3.3)

^1^ Body mass index = weight (kg)/height squared (m^2^).

**Table 2:** Results of the spectral edge frequency 50% of alpha (ASEF50) by electroencephalography, according to the presence and absence of *Streptomyces* *rimosus (S. rimosus)* in the soil during the soil mixing activity.

Soil Mixing Activity	ASEF50 ^1^	
	O1	O2
	Mean ± SD ^2^	
Using soil with *S. rimosus* added after sterilization	10.376 ± 0.137	10.399 ± 0.120
Using sterilized soil	10.350 ± 0.107	10.352 ± 0.121
Significance ^3^	0.132	0.008 **

^1^ ASEF50 is the area from 8 to 13 Hz, which occupies 50% of the area in the entire frequency range. ^2^ SD, standard deviation. ^3^ ** *p* < 0.01 by the paired *t*-test.

## Data Availability

The datasets generated for this study are available on request to the corresponding author.

## Supplementary Materials

These are available online at https://www.mdpi.com/article/10.3390/ijerph191912901/s1, as follows: Table S1: Significantly different VOCs identified by SPME–GC–TOF–MS in soil samples treated through S, SM, and inoculated *S. rimosus*. Table S2: Significantly different serum metabolites between the soil mixing activity groups including control and treatment subjects analyzed via GC–TOF–MS.
